# Vasoproliferative process resembling pulmonary capillary hemangiomatosis in a cat

**DOI:** 10.1186/s12917-017-0984-9

**Published:** 2017-03-20

**Authors:** J.A. Jaffey, K. J. Williams, I. Masseau, M. Krueger, C. Reinero

**Affiliations:** 1 University of Missouri Veterinary Health Center, Columbia, MO USA; 2 Michigan State University, East Lansing, MI USA; 3 Université de Montréal, St-Hyacinthe, Québec Canada; 4 Veterinary Specialty Hopsital of Hong Kong, Wan Chai, Hong Kong

**Keywords:** Pulmonary hypertension, Pulmonary thromboembolism, Computed tomography, Lung, Pulmonary vascular disease, Feline, Ground glass opacity, Pulmonary veno-occlusive disease, Angiography, Animal model

## Abstract

**Background:**

Pulmonary capillary hemangiomatosis is a rare, vascular obstructive disorder that uniformly causes pulmonary arterial hypertension. Clinically, pulmonary capillary hemangiomatosis is indistinguishable from primary pulmonary arterial hypertension and histology is required for definitive diagnosis. The distinctive histologic feature of pulmonary capillary hemangiomatosis is non-malignant extensive proliferation of capillaries in the alveolar septae. Vasodilator treatment of humans with primary arterial hypertension due to pulmonary capillary hemangiomatosis can result in fatal acute pulmonary edema. Computed tomography is thus critical to discern pulmonary capillary hemangiomatosis from other causes of pulmonary arterial hypertension prior to vasodilator therapy. This is the first report of a vasoproliferative process resembling pulmonary capillary hemangiomatosis in the feline species.

**Case presentation:**

A 15-year-old, male castrated, domestic shorthair cat presented for persistent labored breathing presumptively due to congestive heart failure despite treatment with diuretics for 7 days. Echocardiography showed evidence of hypertrophic cardiomyopathy with severe pulmonary hypertension; however, a normal sized left atrium was not consistent with congestive heart failure. Thoracic computed tomography was performed and showed evidence of diffuse ill-defined nodular ground glass opacities, enlarged pulmonary arteries, and filling defects consistent with pulmonary thromboembolism. The cat acutely decompensated after a single dose of sildenafil and was euthanized. Histopathology of the lungs showed severe multifocal alveolar capillary proliferation with respiratory bronchiolar infiltration, marked type II pneumocyte hyperplasia and multifocal pulmonary arterial thrombosis.

**Conclusion:**

This is the first description in a cat of a vasoproliferative disorder resembling pulmonary capillary hemangiomatosis complicated by multifocal pulmonary arterial thrombosis. Inspiratory and expiratory ventilator-driven breath holds with angiography revealed lesions predominantly characterized by ground glass opacification and vascular filling defects with absence of air trapping. The results from this report suggest that, as in humans, the cat can develop a pulmonary capillary hemangiomatosis-like disease in which vasodilator therapy to address pulmonary hypertension may lead to fatal pulmonary edema.

## Background

Pulmonary capillary hemangiomatosis (PCH) is a rare, idiopathic vascular disease that uniformly causes pulmonary arterial hypertension (PAH) [1]. It was first described in humans in 1978 as researchers observed atypical proliferation of capillary-like channels in lung tissue that appeared to be angiomatous growths [1]. The distinctive histologic feature of PCH is the proliferation of capillaries in the pulmonary parenchyma [1, 2]. Additional important features include evidence of invasion by the capillaries into one or more of the pulmonary veins and arteries, alveolar walls and alveolar space, interlobular fibrous septa, and bronchioles [2]. Cytologic atypia and mitoses are absent in PCH lesions [1]. There have been under 100 cases reported in humans in the literature [3]. PCH as a feature of pulmonary venoocclusive disease (PVOD), an obstructive disorder affecting the post-capillary (venous) pulmonary vasculature that has been reported in humans [4] and dogs [5]. To the author’s knowledge PCH has not been described in the cat.

The pathogenesis of PCH is poorly understood and is likely multifactorial. In humans, PCH has been described in association with aortic stenosis [6], Kartagener syndrome [7], systemic lupus erythematous [8], scleroderma [9], Takayasu’s arteritis [10], hypertrophic cardiomyopathy [11] and neoplasia [12, 13]. Mutations in the eukaryotic translation initiation factor 2 alpha kinase 4 (EIF2AK4) gene are a risk factor in the development of PCH [14]. It has also been proposed that PCH is not a separate disease, but rather represents a secondary angioproliferative response to pulmonary venous hypertension as seen in PVOD [4].

The current clinical classification of PH in people has been categorized in five groups of disorders: pulmonary arterial hypertension (Group 1), PVOD and/or PCH (Group 1’), persistent pulmonary hypertension of the newborn (Group 1”), pulmonary hypertension due to left heart disease (Group 2), pulmonary hypertension due to chronic lung diseases and/or hypoxia (Group 3), chronic thromboembolic pulmonary hypertension (Group 4), and pulmonary hypertension due to unclear multifactorial mechanisms (Group 5) [15]. There is a paucity of information regarding PH in cats, mainly being limited to case reports and small case series. Our proposed adaptation of the classification system used in people with PAH to cats based on the available literature is shown in Table 1 [16–38].

**Table 1:** Feline Classification of Pulmonary Hypertension ^a^

1. Pulmonary arterial hypertension	
1.1	Idiopathic PAH (NR)
1.2	Heritable PAH (NR)
1.3	Drug and toxin induced (NR)
1.4	Associated with:
1.4.1	Connective tissue disease (NR)
1.4.2	Immunodeficiency virus (FIV) infection (NR)
1.4.3	Portal hypertension (NR)
1.4.4	Congenital heart diseases: Patent ductus arteriosus [16–18]; Atrial septal defect [19]; Partial anomalous pulmonary venous connection [20];Double-outlet right atrium [37];Ventricular septal defect [38]
1.4.5	Schistosomiasis (NR)
1’ Pulmonary veno-occlusive disease and/or pulmonary capillary hemangiomatosis	
	Current report
1” Persistent pulmonary hypertension of the newborn (NR)	
2. Pulmonary hypertension due to left heart disease	
2.1	Left ventricular systolic dysfunction (NR)
2.2	Left ventricular diastolic dysfunction (NR)
2.3	Valvular disease (NR)
2.4	Congenital/acquired left heart inflow/outflow tract obstruction and congenital cardiomyopathies: Supra valvular mitral stenosis [21–23]; Cor triatriatum [24–28]
3. Pulmonary hypertension due to lung diseases and/or hypoxia	
3.1	Chronic obstructive pulmonary disease
	Nasopharyngeal polyp induced hypoxia [30]
3.2	Interstitial lung disease
	Interstitial pulmonary fibrosis [29]
3.3	Other pulmonary diseases with mixed restrictive and obstructive pattern (NR)
3.4	Sleep-disordered breathing (NR)
3.5	Alveolar hypoventilation disorders (NR)
3.6	Chronic exposure to high altitude (NR)
3.7	Developmental lung diseases (NR)
4. Chronic thromboembolic pulmonary hypertension	
	Pulmonary thromboembolism [31–33]
5. Pulmonary hypertension with unclear multifactorial mechanisms	
5.1	Hematologic disorders: chronic hemolytic anemia (NR), myeloproliferative disorders (NR), splenectomy (NR)
5.2	Systemic disorders: sarcoidosis (NR), pulmonary histiocytosis (NR), lymphangiomyomatosis (NR)
5.3	Metabolic disorders: glycogen storage disease (NR), Gaucher disease (NR), thyroid disorders (NR)
5.4	Others: tumoral obstruction (NR), fibrosing mediastinitis (NR), chronic renal failure (NR), segmental PH (NR)
5.4.1 (proposed in cats)	*Dirofilaria immitis* [34, 35]; *Aelurostrongylus abstrusus* [36]

*NR* not reported, *PAH* pulmonary arterial hypertension, *FIV* feline immunodeficiency virus

^a^ Adapted for the cat from the Human 5th World Symposium on Pulmonary Hypertension Nice, France 2013

The prognosis for PCH in humans is poor, and without lung transplant ultimately fatal. The median survival is approximately 3 years after onset of clinical signs [1, 39]. The only definitive treatment is lung transplantation, though some therapies (i.e. imatinib [40, 41], doxycycline [42], interferon [alpha]-2a [43]) have been shown to improve quality of life and survival time. Clinical signs associated with PCH include progressive dyspnea, exertional fatigue, syncope, and sometimes a chronic cough [1] and are frequently seen in other forms of PAH. The commonality in presenting clinical signs makes differentiating PCH from other forms of PAH challenging. While difficult, this distinction is essential because conventional treatment with vasodilators for PAH is contraindicated in patients with PCH as it can result in fatal pulmonary edema [44–46].

Histopathologic examination of lung tissue for definitive confirmation of PCH requires invasive sampling and can be contraindicated depending on patient stability. In cats, high resolution computed tomography (HRCT) has been used to help identify interstitial lung diseases including interstitial pulmonary fibrosis [29], alveolar filling disorders such as pulmonary alveolar proteinosis [47] and vascular disease like pulmonary thromboemboli [48] and partial anomalous pulmonary venous connection [49]. In humans, HRCT has been used to differentiate PAH from PCH [50]. Main pulmonary arterial enlargement and diffuse ill-defined centrilobular nodules of ground glass opacity are commonly seen in people with PCH but the latter are not seen with PAH [1, 51]. With increased recognition of other causes of pulmonary hypertension, and in particular PAH in the cat, future use of HRCT may be an invaluable diagnostic for pulmonary hypertension in Groups 1, 3, and 4 (see Table 1). Importantly, prior to initiating vasodilator therapy, HRCT may allow differentiation of PCH and PVOD from PAH in veterinary species as it does in humans [1, 51].

## Case presentation

A 15-year old male castrated domestic shorthair cat was referred to the University of Missouri Veterinary Health Center for evaluation of presumed congestive heart failure. The cat was clinically healthy until the age of 14 years, when he experienced an episode of abrupt, severe fatigue while playing with a toy. The cat did not lose consciousness and recovered quickly with no medical intervention. The cat remained clinically normal for approximately a year, at which time he suffered a brief syncopal event while playing with a toy. The cat was evaluated by the referring veterinarian and treated with furosemide^a^ (2.2 mg/kg, PO, q12 hr) for presumptive congestive heart failure. The cat subsequently developed a decreased appetite and an increase in respiratory effort and rate until presentation 7 days later.

The cat was the only animal in the house and never ventured outdoors. The owner did not have any known potentially hazardous hobbies (i.e. painting, crafts, furniture restoration). There was no known exposure to noxious/irritant inhalational agents (i.e. cigarette smoke, air-freshener, incense, etc.). The cat was up to date on vaccinations as well as heartworm and flea preventative.

At presentation the cat was bright, alert, and responsive. He showed tachypnea (60 breaths/minute) with increased inspiratory effort. He was mildly hypothermic (rectal temperature 99°F), and was approximately 5% dehydrated. His heart rate was 160 beats/minute with an occasional irregular rhythm that resulted in pulse deficits. Thoracic auscultation revealed a grade III/VI left parasternal systolic murmur and normal respiratory sounds. The hair coat appeared dull and unkempt. The cat weighed 5.6 kg (12 lbs). The rest of the physical examination was unremarkable.

**Fig. 1:**
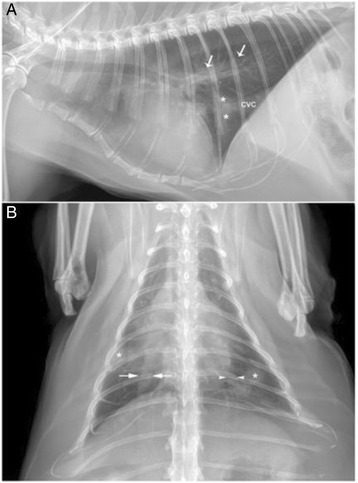
Orthogonal radiographic projections of a 15-year-old cat with pulmonary capillary hemangiomatosis. **a** Left lateral projection showing mild cardiomegaly, the cardiac silhouette occupying almost three intercostal spaces. The caudal pulmonary vessels are ill-defined and less opaque than normally expected (*arrows*). A mild focal unstructured interstitial pattern (*) is seen in the ventral aspect of the caudal lung field, partly overlying the caudal vena cava (CVC). **b** Dorsoventral projection demonstrating mild cardiomegaly with the cardiac silhouette occupying more than 50% of the hemithorax measured at the ninth ribs. The caudal lobar pulmonary arteries are larger than the corresponding pulmonary veins measured at the ninth ribs (*right*: between *arrows*, *left*: between *arrowheads*). The left caudal lobar pulmonary artery abruptly becomes less distinct caudal to the ninth rib. Multifocal patchy areas of unstructured interstitial pattern with ill-defined borders (*) are seen in the *right* and *left* caudal lung lobes

Thoracic radiographs showed cardiomegaly and mild enlargement of the right pulmonary artery. The left pulmonary was larger than the left pulmonary vein proximally but became ill defined at the level of the 9th rib. In addition, there was a multi-focal interstitial pattern within the ventral aspect of caudal lung lobes (Fig. 1). Serum antigen for feline heartworm and feline leukemia virus and serum antibodies for feline immunodeficiency virus were not detected.^b^ Serum total T4 concentration was normal (2.3ug/dL; reference interval, 0.8 to 4.0 ug/dL). Serum biochemical parameters outside of the reference interval were glucose (178 mg/dL; reference interval 52 to 153 mg/dL), blood urea nitrogen (59 mg/dL; reference interval 17 to 35 mg/dL), creatinine (3.1 mg/dl; reference interval 0.5 to 2.2 mg/dL), and potassium (2.5 mEq/L; reference interval 3.0 to 4.7 mEq/L). Hematologic abnormalities were neutrophilia (13.34 × 10^3^/uL; reference interval, 2.5 to 12.5 × 10^3^/uL), lymphopenia (1.48 × 10^3^/uL; reference interval, 1.5 to 7.0 × 10^3^/uL), and monocytosis (1.15 × 10^3^/uL; reference interval, 0 to 0.85 × 10^3^/uL). A urinalysis obtained via cystocentesis showed a urine specific gravity of 1.019. The hematologic abnormalities and mild hyperglycemia were believed to be secondary to stress. The hypokalemia, azotemia and minimally concentrated urine were presumed to be secondary to diuretic therapy; however chronic kidney disease could not be ruled out as there was no serum biochemical panel or urinalysis available for comparison prior to initiation of diuretic therapy. Abdominal ultrasonography was unremarkable.

**Fig. 2:**
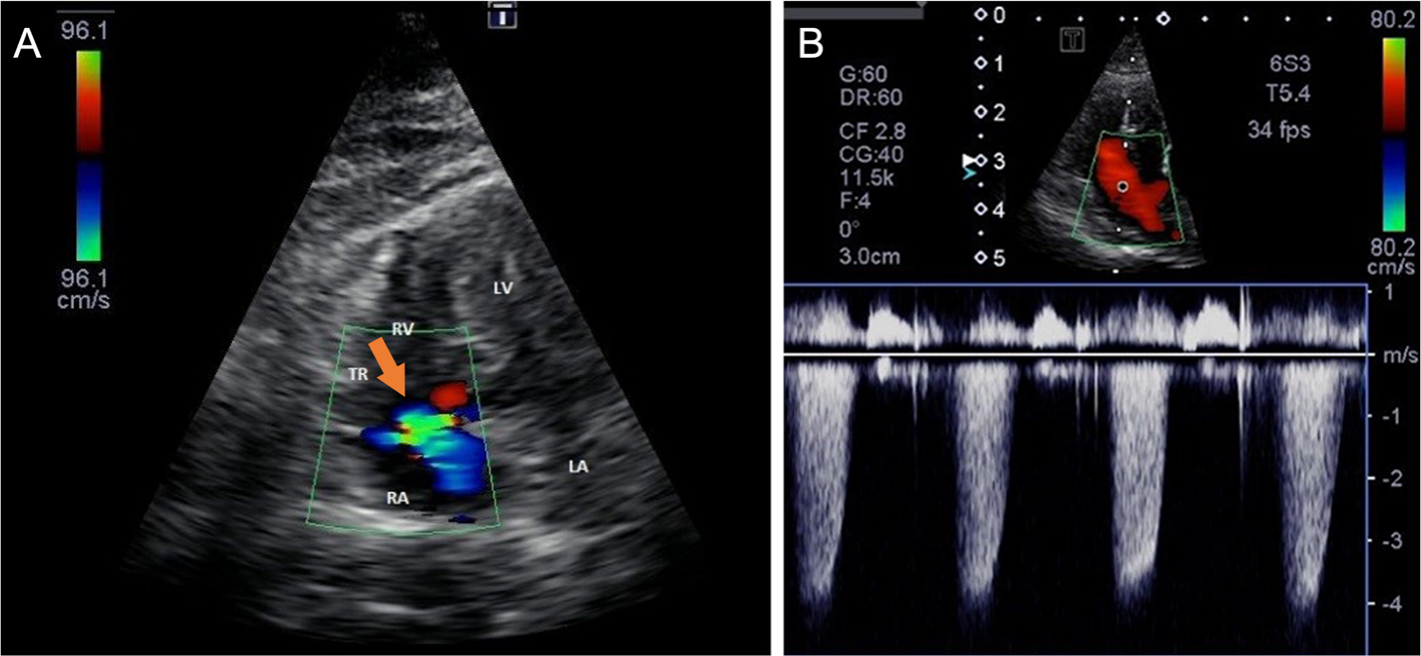
Tricuspid regurgitation. Color Doppler image of tricuspid regurgitation (**a**) and Continuous Doppler (CW) image (**b**) from the left apical 4-chamber view optimized for the right ventricle. Tricuspid regurgitation approximates 4.2m/sec, indicating a peak tricuspid regurgitation pressure gradient of approximately 70mmHg (moderate pulmonary hypertension). LA—left atrium, LV—left ventricle, RA—right atrium, RV—right ventricle, TR—tricuspid regurgitation (*orange arrow*)

**Fig. 3:**
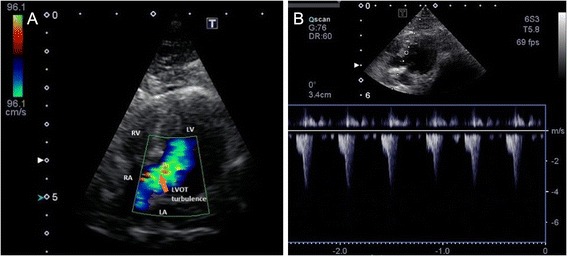
Turbulent flow in the left ventricular outflow tract. Color Doppler image (**a**) and Continuous Doppler image (**b**) both acquired from the left apical 5-chamber view. Note the acceleration of LVOT flow in end-systole. LA—left atrium, LV—left ventricle, RA—right atrium, RV—right ventricle, LVOT—left ventricular outflow tract (*orange arrow*)

The echocardiogram revealed significant left ventricular hypertrophy, moderate right ventricular hypertrophy and normal left atrial dimensions (Fig. 2a-b). Color Doppler Echocardiography showed a moderate amount of tricuspid regurgitation (Fig. 3a). Continuous-wave Doppler assessment of the peak tricuspid regurgitation was measured at 4.2m/sec resulting in a tricuspid regurgitation pressure gradient between the right atrium and right ventricle of 70.2mmHg (calculated using the modified Bernoulli equation, ∆p = 4V^2^)(Fig. 3b). Due to the tricuspid regurgitation jet direction the true peak velocity and with that the pressure gradient may have been underestimated. By adding the estimated right atrial pressure, 75.2mmHg was calculated as the systolic pulmonary artery pressure [52]. Due to the lack of a pulmonary arterial pressure classification scheme specific for cats, this information was extrapolated from the dog [53]. The cat was subsequently diagnosed with hypertrophic obstructive cardiomyopathy and moderate to severe pulmonary arterial hypertension (PAH). Given the normal left atrial size it was concluded that congestive heart failure was unlikely. Based on echocardiography a primary cause for PAH was not identified. There was no evidence of structures consistent with adult *D. immitis* or thromboemboli in the main pulmonary artery or in close proximity to the bifurcation. However, their presence could not be excluded further distally.

The cat was housed in an oxygen chamber and treated overnight with 40% inhalational oxygen and intravenous fluids. While being treated with supplemental oxygen, the cat’s respiratory effort normalized and rate decreased.

The following day the cat had thoracic computed tomography (CT, 64-detector row Toshiba Aquilion, Toshiba America Medical Systems, Tustin, CA) to determine an identifiable underlying cause of PH (i.e. primary lung disease or thromboemboli). The cat was premedicated with butorphanol^b^ (0.5 mg/kg IV). Following premedication, general anesthesia was induced with alfaxalone^c^ (1.8 mg/kg IV) and maintained with intravenous alfaxalone (0.2 mg/kg/min). The cat was intubated and mechanically ventilated (Engstrom Carestation ventilator, GE Healthcare). The ventilator settings included volume-controlled ventilation with an inspired oxygen concentration of 40%, tidal volume 10 mls/kg, respiratory rate 10 breaths/minute, and positive end-expiratory pressure (PEEP) 5 cm H_2_0. Inspiratory and expiratory breath holds were ventilator-assisted and performed in tandem with CT scans; PEEP was set to 0 cm H_2_0 for the expiratory breath hold.

**Fig. 4:**
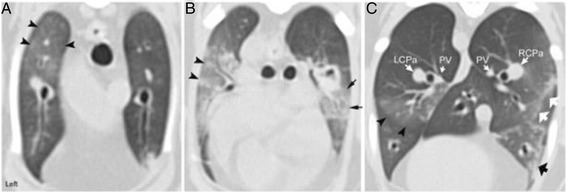
Inspiratory breath-hold transverse computed tomographic images of a 15-year-old cat with pulmonary capillary hemangiomatosis. **a** Multiple small ground glass opacities are seen in the cranial part of the left cranial lung lobe (*arrowheads*). **b** Patchy areas of ground glass opacification appearing as nodular in some areas (*arrowheads*) progressing towards consolidation medially in the cranial part of the left cranial lung lobe. Hazy ground glass opacification is also present in the ventral aspect of the right cranial lung lobe (*arrows*). **c** Ground glass opacification is noted along the ventral margin of the left caudal lung lobe (*arrowheads*). The fissure line at the intersection of the right caudal and right middle lung lobes is thickened and irregular (*white arrows*). Subpleural thickening is also observed in the right middle lung lobe (*black arrow*). The left (LCPa) and right (RCPa) caudal lobar pulmonary arteries are larger than their counterpart pulmonary veins (PV)

**Fig. 5:**
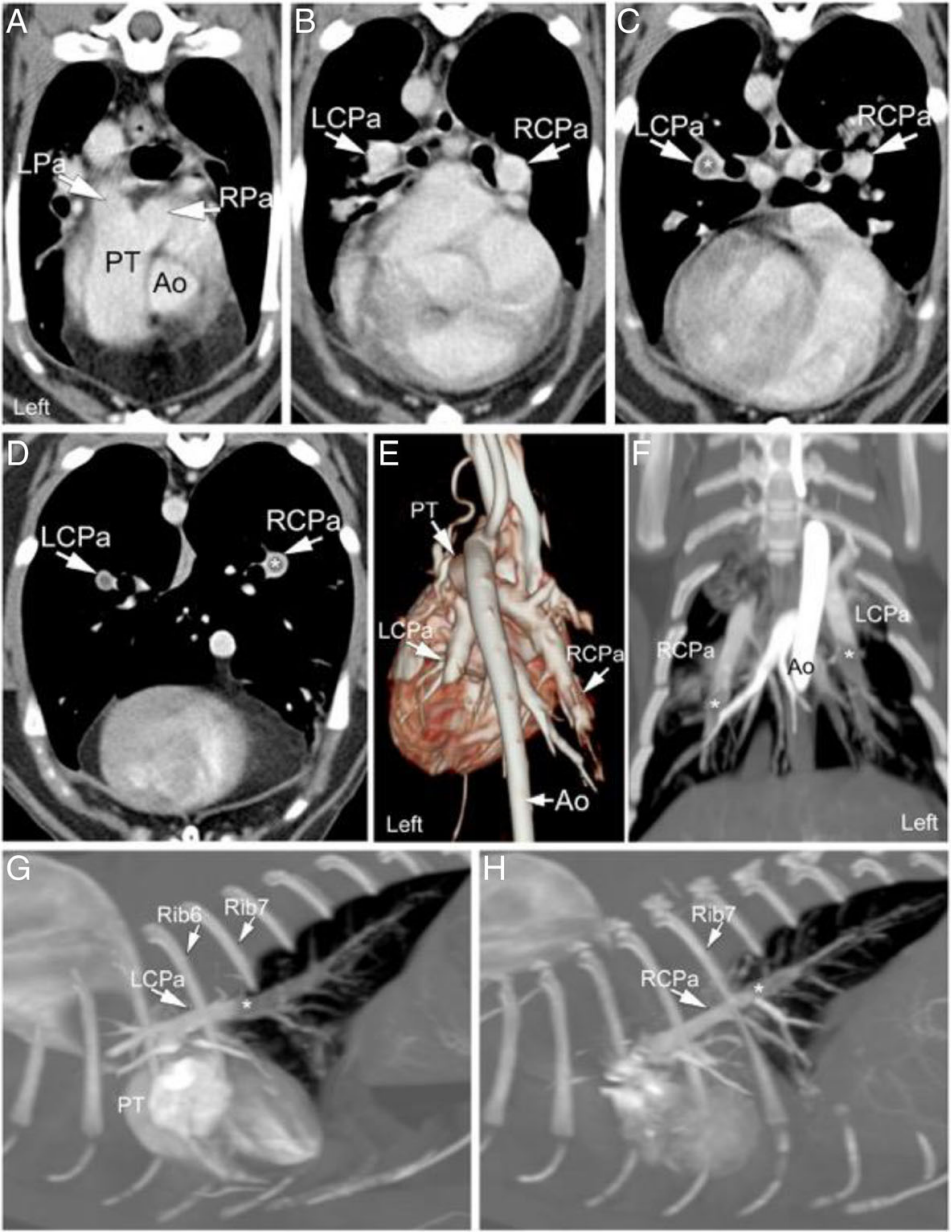
Pulmonary arterial enlargement and thromboembolism in a 15-year-old cat with pulmonary capillary hemangiomatosis. **a**-**d** Transverse post-contrast CT images extending from the level of the pulmonary trunk to the level of T9. **a** The pulmonary trunk (PT) is enlarged displacing the aorta (Ao) to the right. The pulmonary trunk as well as the left (LPa) and right (RPa) pulmonary arteries are filled with iodinated contrast medium. **b** After their entry into the hilus of the lungs and division of the first branches, the right and left pulmonary arteries now becoming the caudal lobar pulmonary arteries (LCPa, RCPa) still present good filling of the entire lumen of the arteries with contrast medium. **c** After giving off a branch to the caudal segment of the left cranial lung lobe, the left caudal lobar pulmonary artery (LCPa) presents a large filling defect (*) encompassing the entire lumen of the vessel while no filling defect is identified in the right caudal lobar pulmonary artery (RCPa) at this level (8th thoracic vertebra). **d** After giving off a branch to the ventral aspect of the right caudal lung lobe, a large filling defect (*) involving the entire lumen of the RCPa is seen. **e** Dorsal projection of the heart and great vessels obtained using a three-dimensional (3D) volume rendering application tool of the CT workstation based on volume data from the post-contrast arterial phase. The lack of enhancement due to the absence of contrast medium first in the LCPa followed approximately 1 cm caudally by the right (RCPa) resulted in an abrupt termination of those vessels instead of a normal tapering towards the periphery of the lung lobes. Horizontal (**f**) and sagittal (G-H) post-contrast 3D Maximum Intensity Projection images of the left (**g**) and right (**h**) caudal lobar pulmonary arteries illustrating the location of the filling defects in relation to the longitudinal path of these vessels. On the left side, the artery (LCPa) abruptly lacks contrast enhancement (*) starting between the 6th (Rib6) and 7th ribs (Rib7) and throughout the caudal extent of the vessel. On the right side, the lack of contrast enhancement in the artery starts just caudal to the 7th rib and over a length of 4 mm, a filling defect (*) encompassing the entire lumen and throughout the remainder of the artery is illustrated

Contiguous 1–2 mm transverse images, pre-and post-contrast administration, were obtained from the level of C6 to L2 with the cat in sternal recumbency. Both inspiratory and expiratory sequences were performed as well as arterial, venous, and delayed phases. Iodinated contrast^d^ was injected at 3 ml/s velocity with a total volume of 12 ml (2.1 mg/kg). The sure-start feature of the CT scanner was used to automatically start image acquisition when the threshold of 180 Hounsfield units (HU) inside the aortic-arch was reached thus defining the arterial phase. The image acquisition of the venous phase started automatically following termination of the arterial phase. The images acquired in the delayed phase were obtained exactly 2 min following the start of the arterial phase. On the pre-contrast inspiratory sequence, there were multiple patchy areas of ground glass opacification in the lung lobes especially in the cranial lobes that were of similar size and equally distant from each other (Fig. 4a). The most peripheral opacities were all at the same distance from the pleural surface. Ground glass opacification was also seen in the transition between areas of consolidation and normal lung attenuation in the ventral aspects of the lungs bilaterally sparing only the most cranial and most caudal portions of the lungs (Fig. 4b-c). The left and right caudal lobar pulmonary arteries were enlarged, being larger than the corresponding vein (Fig. 4c). In the post-contrast inspiratory venous sequence, the pulmonary trunk was subjectively enlarged (1.11 cm vs. 0.929 cm ascending aorta), the ascending aorta was displaced to the right by the enlarged right ventricular outflow tract/pulmonary trunk (Fig. 5a). The post-contrast arterial phase showed the presence of iodinated contrast medium in the pulmonary trunk, left and right pulmonary arteries (Fig. 5a) and the proximal aspect of the caudal lobar pulmonary arteries (Fig. 5b). Starting at the level of the 6th ribs on the left (Fig. 5c) and of the 7th ribs on the right (Fig. 5d), both caudal lobar pulmonary arteries demonstrate a major filling defect occupying the entire lumen of the artery. The lack of contrast medium in the caudal lobar pulmonary arteries is well illustrated by the abrupt termination of these arteries in relation to the pulmonary trunk and thoracic aorta on the three-dimensional (3D) volume rendering image (Fig. 5e). 3D Maximum Intensity Projection tool available on the CT workstation was used to elongate each artery in the horizontal (Fig. 5f) and sagittal (Fig. 5g-h) planes providing excellent visualization of the filling defects in relation to the length of each artery.

Following the CT, the cat was maintained in a light plane of anesthesia with 5 to 6 cm H_2_0 PEEP for an additional hour until he was able to maintain an oxygen hemoglobin-saturation above 95% without PEEP. The cat then recovered in an oxygen chamber providing 40% inhalational oxygen. The cat continued to show tachypnea, maintained oxygen enriched hemoglobin-saturations of 90 to 95%, and had clear lung sounds on thoracic auscultation.

Based on diagnostics performed at that time it was suspected the cat had a significant pulmonary vasculopathy that resulted in PAH. The etiology of the vasculopathy was unknown at that time but suspected secondary to thromboembolic disease as well as uncharacterized primary lung disease. The cat was treated with one dose of sildenafil^d^ (0.9mg/kg, PO). Approximately 45 min following administration of sildenafil the cat developed respiratory distress. His respiratory rate increased to 80 breaths/min, he assumed an orthopneic posture, and was breathing with an open mouth. On thoracic auscultation the cat had harsh, loud crackles in all lung fields.

Because of the progressively worsening respiratory distress as well as the grave prognosis, the owner elected euthanasia. Immediately after euthanasia the lungs were removed with owner consent and fixed for histopathology. In an effort to prevent the collapse, deflation, and disruption of lung structures and to avoid fixation artifacts, each lung lobe was inflation-fixed with 10% formalin [54]. Each lobar bronchus was cannulated with a 3 French red rubber catheter and fixed with a ligature. The lungs were inflated via the cannula by gentle infusion of 10% formalin. After each lobe was sufficiently inflated, the lobar bronchus was tied off with a ligature and the lungs were placed in jars containing 10% formalin (ratio at least 1 part tissue to 9 parts formalin).

**Fig. 6:**
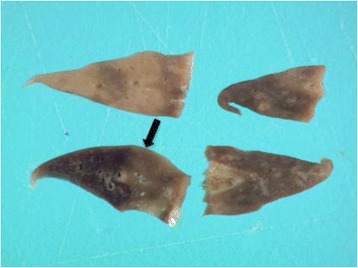
Subgross fixed lung lobes obtained post-mortem in a 15-year-old cat with pulmonary capillary hemangiomatosis. Sharply demarcated dark red foci indicative of the vascular lesions were evident within the lung parenchyma adjacent to more normal (*black arrow*) regions of fixed lung

**Fig. 7:**
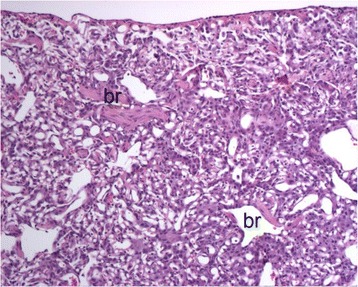
Lung histopathology in a cat with PCH-like lung disease. The alveolar parenchyma in foci of affected lung is comprised of numerous capillaries that obscure the normal alveolar architecture and surround and infiltrate into bronchioles (br). Hematoxylin-eosin stain

**Fig. 8:**
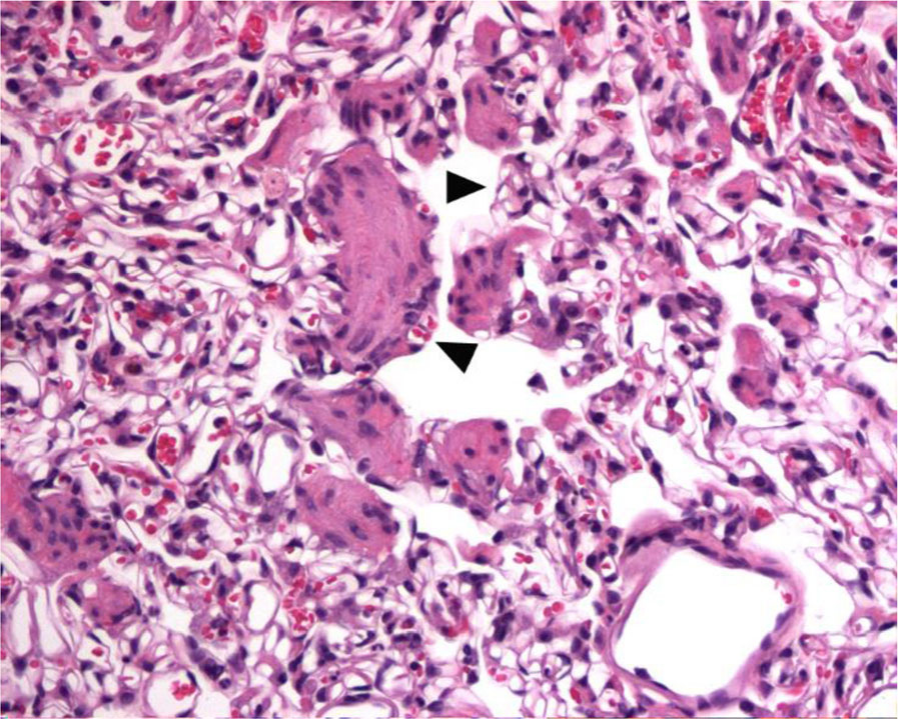
Lung histopathology in a cat with PCH-like lung disease. Capillary profiles (*arrowheads*) are present between and surrounding the bundles of smooth muscle of a respiratory bronchiole. Hematoxylin-eosin stain

**Fig. 9:**
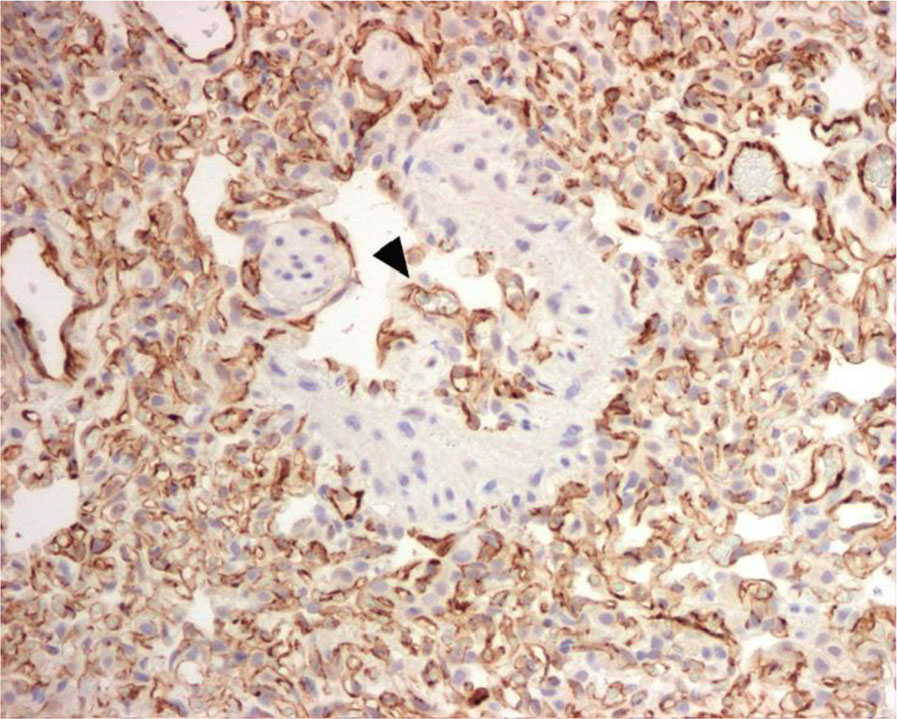
Lung immunohistochemistry for expression of the endothelial cell surface antigen CD31 in a cat with PCH-like disease. Antibody against CD31 identifies capillary endothelial cells infiltrating around bronchiolar smooth muscle (*arrowhead*) as well as highlighting the increased numbers of capillaries within the surrounding alveolar parenchyma. Immunohistochemistry, diaminobenzidine chromagen

In the subgross fixed lung lobes multifocal sharply demarcated dark red foci were evident within the lung parenchyma (Fig. 6). Histopathology of these regions were characterized by severe multifocal alveolar capillary proliferation (Figs. 7, 8 and 9). Foci of infiltration of respiratory bronchioles was frequent and marked type II pneumocyte hyperplasia was present in the remodeled lung. In addition, there was moderate focally extensive alveolar congestion with intra-alveolar hemorrhage and fibrin. Furthermore, there was evidence of multifocal pulmonary arterial thrombosis and alveolar carcinomas. The apparent capillary proliferation with infiltration of respiratory bronchioles is suggestive of PCH. Although PCH often occurs along with PVOD, there was no evidence of PVOD in this cat. The epithelial neoplasms were considered to be incidental findings in this case.

Given the timing of the sildenafil to the cat’s acute respiratory decompensation (45 min prior) it is suspected that arteriolar dilation with a fixed capillary obstructive lesion led to flooding of the alveoli from increases in hydrostatic pressure. In humans with post-capillary PAH (i.e. PCH and PVOD) treatment with vasodilators can result in a florid and fatal pulmonary edema. It is also possible the cat’s abrupt respiratory decline included additional acute pulmonary thromboemboli; anaphylactic response to the compounded sildenafil or the onset of acute respiratory distress syndrome were not supported by histopathologic findings.

The cause for thromboembolic disease was not determined but could have been secondary to cardiac disease. Interestingly, a recent report evaluating the microcirculation in people with chronic thromboembolic pulmonary hypertension found a strong association with capillary hemangiomatosis-like lesions [55].

## Discussion and conclusions

Pulmonary capillary hemangiomatosis is a rare, idiopathic angioproliferative disease that results in PAH [1]. The unique histologic feature of PCH is the proliferation of capillaries in the pulmonary parenchyma as well as evidence of invasion by the capillaries into one or more of the pulmonary veins, arteries, alveolar walls, alveolar space, interlobular fibrous septa, and bronchioles [2]. Histologic evaluation of lung parenchyma from the cat in this report revealed severe multifocal alveolar capillary proliferation with infiltration of respiratory bronchioles suggestive of PCH. The pathogenesis of PCH is poorly understood and is likely multifactorial. In humans, PCH has been described in association with several disease processes includingaortic stenosis, Kartagener syndrome, systemic lupus erythematous, scleroderma, Takayasu’s arteritis, hypertropic cardiomyopathy and neoplasia [6–13]. Echocardiographic evaluation of the cat reported here revealed significant left ventricular hypertrophy and normal left atrial dimensions. In humans, it is postulated that long standing chronic passive congestion of the lung as is seen with hypertrophic cardiomyopathy results in development of PCH [11]. The normal left atrial dimensions in this cat make chronic pulmonary venous congestion unlikely. It has also been proposed that PCH is not a separate disease, but rather represents a secondary angioproliferative response to pulmonary venous hypertension as seen in PVOD [4]. However, there was no evidence of PVOD in this cat. In humans, mutations in the EIF2AK4 gene are a risk factor in the development of PCH [14]. Future studies investigating the aforementioned EIF2AK4 gene mutation are needed to establish this association in dogs and cats with PCH.

Pulmonary hypertension is a syndrome characterized by altered blood flow resulting in elevated pulmonary arterial pressures. In veterinary medicine, PH is classified based on the origin of altered blood flow, that is, pre-capillary (pulmonary arterial hypertension) or post-capillary (pulmonary venous hypertension [56]. The clinical picture was complicated by pulmonary thromboembolism, which also contributed to PH. Thus the PH identified in the cat described in this report would likely be classified as both Group 1’ and Group 4 (Table 1).

In humans, clinical signs associated with PCH include progressive dyspnea, exertional fatigue, syncope, and sometimes a chronic cough [1] and are frequently seen in other forms of PAH. The commonality in presenting clinical signs makes differentiating PCH from other forms of PAH challenging. While difficult, this distinction is essential because conventional treatment with vasodilators for PAH is contraindicated in patients with PCH as it can result in fatal pulmonary edema [44–46]. It is currently recommended that patients with presumed primary pulmonary hypertension undergo a HRCT examination before initiation of vasodilator therapy [1]. The CT in this case corroborates common CT findings in humans with PCH; diffuse, ill-defined nodular ground glass opacities and enlarged pulmonary arteries. Given the timing of the sildenafil to the cat’s acute respiratory decompensation (45 min prior) it is suspected that arteriolar dilation with a fixed capillary obstructive lesion led to flooding of the alveoli from increases in hydrostatic pressure.

## Abbreviations

CT: Computed tomography
HRCT: High resolution computed tomography
HU: Hounsfield units
PAH: Pulmonary arterial hypertension
PCH: Pulmonary capillary hemangiomatosis
PEEP: Positive end-expiratory pressure
PVOD: Pulmonary venoocclusive disease
